# Early and Late Effects of Low-Dose X-ray Exposure in Human Fibroblasts: DNA Repair Foci, Proliferation, Autophagy, and Senescence

**DOI:** 10.3390/ijms25158253

**Published:** 2024-07-28

**Authors:** Andrey Osipov, Anna Chigasova, Elizaveta Yashkina, Maxim Ignatov, Natalia Vorobyeva, Nikolay Zyuzikov, Andreyan N. Osipov

**Affiliations:** 1 N.N. Semenov Federal Research Center for Chemical Physics, Russian Academy of Sciences, 119991 Moscow, Russia; a-2-osipov@yandex.ru (A.O.); annagrekhova1@gmail.com (A.C.); yashkinaliz@gmail.com (E.Y.); mantroz@yandex.ru (M.I.);; 2 Emanuel Institute for Biochemical Physics, Russian Academy of Sciences, 119334 Moscow, Russia; 3 State Research Center—Burnasyan Federal Medical Biophysical Center of Federal Medical Biological Agency (SRC—FMBC), 123098 Moscow, Russia; 4 Department of Physics, Faculty of Science and Technology, The University of the West Indies, St. Augustine 999183, Trinidad and Tobago; nikolay.zyuzikov@sta.uwi.edu; 5 CANDLE Synchrotron Research Institute, 31 Acharyan, Yerevan 0040, Armenia

**Keywords:** DNA double-strand breaks, DNA damage response, DNA repair foci, proliferation, autophagy, senescence, fibroblasts, X-ray radiation, low-dose effects

## Abstract

The effects of low-dose radiation exposure remain a controversial topic in radiation biology. This study compares early (0.5, 4, 24, 48, and 72 h) and late (5, 10, and 15 cell passages) post-irradiation changes in γH2AX, 53BP1, pATM, and p-p53 (Ser-15) foci, proliferation, autophagy, and senescence in primary fibroblasts exposed to 100 and 2000 mGy X-ray radiation. The results show that exposure to 100 mGy significantly increased γH2AX, 53BP1, and pATM foci only at 0.5 and 4 h post irradiation. There were no changes in p-p53 (Ser-15) foci, proliferation, autophagy, or senescence up to 15 passages post irradiation at the low dose.

## 1. Introduction

The effects of low-dose radiation are the subject of intense debate among radiobiologists. On one hand, there are epidemiological studies indicating an increase in the risks of leukemia and brain cancer in children after computed tomography (CT) scans [1,2]. On the other hand, there are numerous studies indicating the positive effects of CT, for example, reducing mortality from lung cancer in humans [3,4,5,6,7]. These studies show the inconsistency of the linear no-threshold (LNT) model, which is based on a linear extrapolation of the effects of high doses of radiation from those of low doses and the existence of a number of poorly understood aspects that require serious fundamental research. 

Among the various types of DNA damage caused by ionizing radiation, double-strand breaks (DSBs) are the most deleterious. They trigger reactions that affect almost every aspect of cellular metabolism and are collectively known as the DNA damage response (DDR) [8]. Unlike single-stranded DNA breaks, which can be easily repaired using a complementary DNA strand, accurate repair of double-strand DNA breaks is often problematic because it requires a template in the form of sister chromatid DNA. In addition to the loss of genetic information, DNA DSBs can lead to fragmentation and rearrangements of chromosomes [9], inactivation of tumor suppressor genes, or activation of proto-oncogenes [10]. Thus, errors in DNA DSB repair can lead to genomic instability and the malignant transformation of cells [11]. Incorrect repair of DSBs formed near telomeres can also lead to their shortening and accelerated replicative senescence of irradiated cells [12]. There is an opinion that mammalian cells cannot provide effective repair of DNA DSBs induced by low-dose radiation due to the lack of induction of G2/M checkpoint arrest and the presence of restrictions on the access of components of DSB repair systems to DNA within heterochromatin [13]. Evidence of the lack of DNA DSB repair in resting human fibroblasts exposed to a very low X-ray dose (1 mGy) was reported [14]. It is also believed that DNA DSB repair is triggered only after a certain DSB threshold is reached [15]. The question arises: if this is indeed the case, can low-dose radiation cause detrimental late effects in the descendants of irradiated cells?

Currently, the most informative and sensitive approach for studying DNA DSBs is the immunocytochemical analysis of proteins involved in the DDR [16]. Dynamic microstructures consisting of hundreds and thousands of different proteins involved in the DDR and localized in regions adjacent to DNA DSBs are called DNA damage foci [17,18] or DNA repair foci [19,20]. The most commonly studied focus-forming proteins are histone H2AX phosphorylated at serine 139 (γH2AX) [21,22,23], 53BP1 (p53-binding protein 1) [24,25,26], and ATM kinase (ataxia telangiectasia mutant protein) phosphorylated at serine 1981 (pATM) [27,28].

The goal of this work was to comprehensively study DNA repair foci, proliferation, autophagy, and senescence in human fibroblasts directly exposed to low-dose X-ray radiation and their descendants (up to the 15th cell passage after irradiation).

Fibroblasts were the cell type of choice in this study because they play an important role in the development of various radiation pathologies. Depending on the fate of irradiated fibroblasts, various negative effects can develop in tissues, including inflammation, fibrosis, and tissue dysfunction [29,30,31]. Potentially, altered irradiated fibroblasts can more easily transform into cancer-associated fibroblasts, which play an important role in cancer progression [32] and resistance to anticancer therapy [33,34].

## 2. Results

### 2.1. Early Effects

#### 2.1.1. DNA Repair Foci

**Figure 1:**
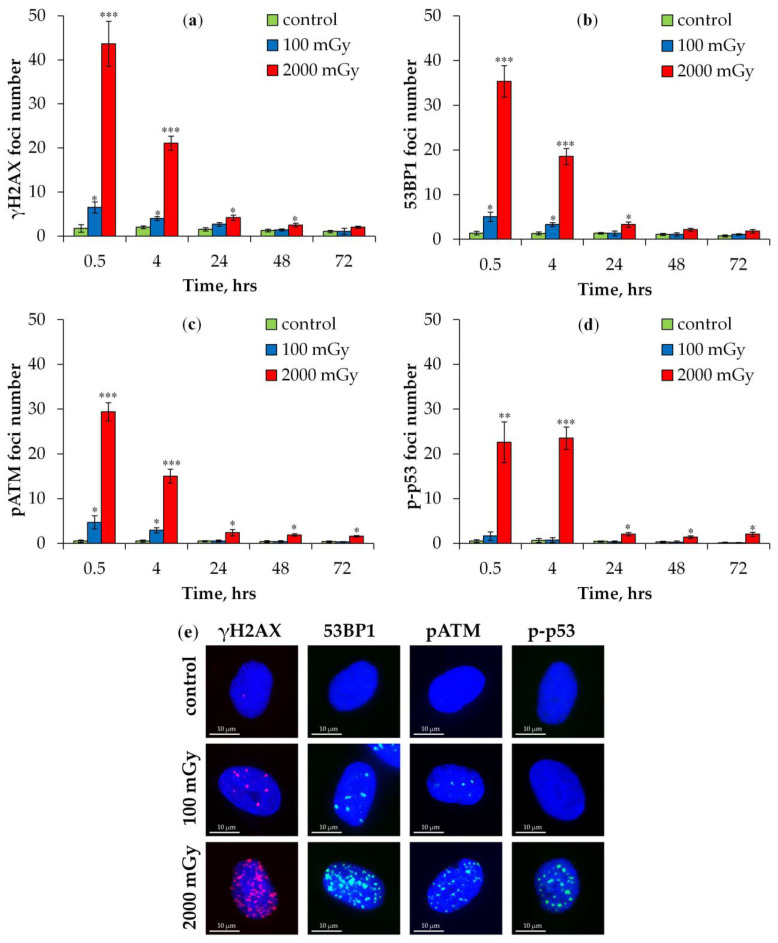
Changes in the number of DNA repair foci in fibroblasts exposed to X-ray radiation at doses of 100 and 2000 mGy: (**a**) γH2AX; (**b**) 53BP1; (**c**) pATM; and (**d**) p-p53. * *p* < 0.05, ** *p* < 0.01, and *** *p* < 0.001 compared with corresponding control. (**e**) Representative microphotographs of immunofluorescently stained cell nuclei of fibroblasts post irradiation (2000 mGy, 0.5 h) showing γH2AX (red), 53BP1 (green), pATM (green), and p-p53 (green) foci. DAPI counterstaining is shown in blue.

The study’s results on post-irradiation changes in the number of DNA repair foci in irradiated human fibroblasts are shown in Figure 1. The representative unprocessed microphotographs of immunocytochemically stained control and irradiated fibroblasts are shown in Figures S1–S4. The maximum number of γH2AX, 53BP1, and pATM foci was observed 0.5 h after irradiation (Figure 1a–c). A total of 43.7 ± 5.1, 35.3 ± 1.9, and 29.4 ± 2.0 foci/nuclei for γH2AX, 53BP1, and pATM, respectively, were counted after irradiation at a dose of 2000 mGy. The 100 mGy exposure induced 6.5 ± 1.3, 5.1 ± 1.0, and 4.7 ± 1.5 foci/nuclei for γH2AX, 53BP1, and pATM, respectively. Control values were 1.8 ± 0.9, 1.4 ± 0.4, and 0.5 ± 0.3 foci/nuclei for γH2AX, 53BP1, and pATM, respectively.

When discussing changes in the absolute values of foci numbers, control values and relative values per dose unit are not considered. We adjusted it for control values and recalculated based on a dose of 1000 mGy. Thus, for a dose of 100 mGy, the foci yields were approximately 48, 37, and 42 foci/Gy for γH2AX, 53BP1, and pATM, respectively. However, for a dose of 2000 mGy, the foci yields were much lower: about 21, 17, and 15 foci/Gy for γH2AX, 53BP1, and pATM, respectively. Therefore, the relative number of foci per dose unit decreased with increasing radiation dose. Four hours after irradiation with a 100 mGy dose, the number of radiation-induced foci decreased by approximately 2.4, 1.9, and 1.8 times compared to those that were induced 0.5 h post irradiation. A similar pattern was observed four hours after irradiation at a dose of 2000 mGy in which the number of radiation-induced γH2AX, 53BP1, and pATM foci decreased by approximately 2.2, 2.0, and 2.0 times, respectively. At 24 h post irradiation, a statistically significant increase in the number of foci was only noted at a dose of 2000 mGy (~6–7% of the number at 0.5 h post irradiation). From 24 to 72 h post irradiation, the number of foci continued to slowly decrease (Figure 1a–c).

A very different foci dynamic for p-p53 was observed (Figure 1d). First, there was no statistically significant foci increase post irradiation at a dose of 100 mGy. Second, the number of foci did not change between 0.5 and 4 h.

**Figure 2:**
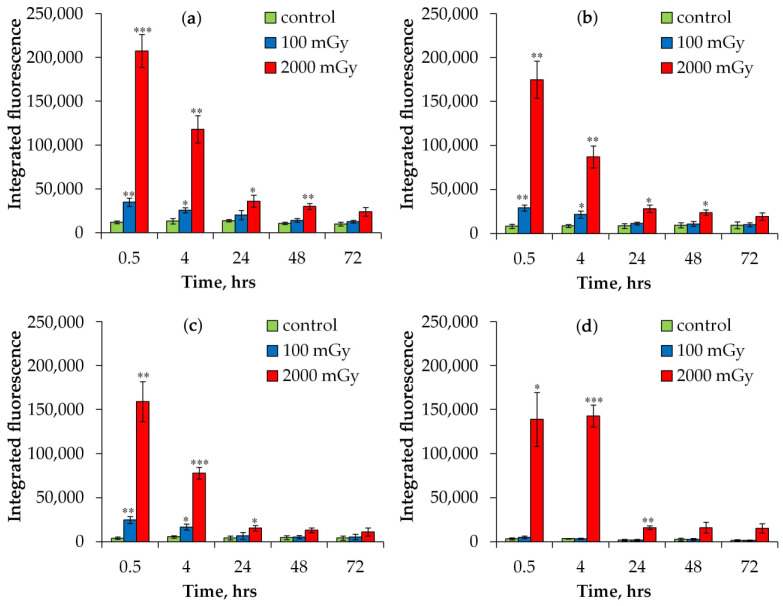
Changes in the integrated fluorescence of immunofluorescently stained DNA repair foci in fibroblasts exposed to X-ray radiation at doses of 100 and 2000 mGy: (**a**) γH2AX; (**b**) 53BP1; (**c**) pATM; and (**d**) p-p53. * *p* < 0.05, ** *p* < 0.01, and *** *p* < 0.001 compared with corresponding control.

As the number of DNA DSBs increases with dose, the probability that two or more breaks will occur in close proximity also increases. Accordingly, several nearby foci can be counted as one focus. This may lead to an underestimation of the real number of foci. In order to avoid this, we measured the integrated fluorescence of foci in control and irradiated cells. As can be seen from the results presented in Figure 2, changes in the value of the integrated fluorescence of foci correlated very well with changes in foci number and confirmed the conclusion made based on the analysis of foci number. According to the results, 0.5 h after irradiation with a dose of 100 mGy, the relative values of the integrated fluorescence of γH2AX, 53BP1, and pATM foci for 1000 mGy were more than two times higher compared to the values at a dose of 2000 mGy: 2.3 times for γH2AX foci; 2.5 times for 53BP1 foci; and 2.7 times for pATM foci. 

#### 2.1.2. Proliferation, Senescence, and Autophagy

**Figure 3:**
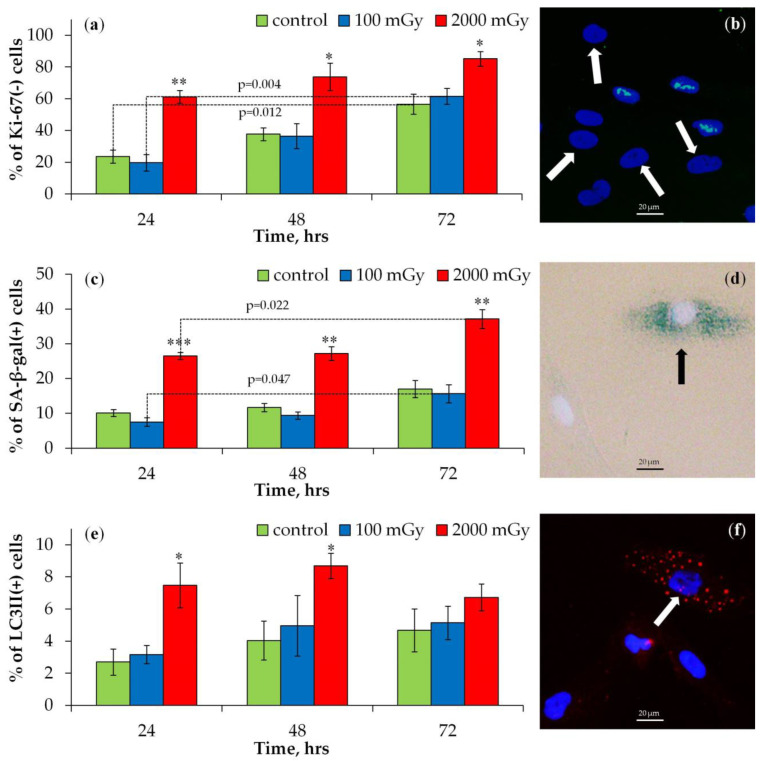
Changes in the proportions of (**a**) Ki-67(-), (**c**) SA-β-gal(+), and (**e**) LC3II(+) cells 24, 48, and 72 h after irradiation at doses of 100 and 2000 mGy. * *p* < 0.05, ** *p* < 0.01, *** *p* < 0.001 compared with corresponding control. (**b**) Representative microphotograph of the immunocytochemically labeled cells (2000 mGy, 8 h) with the Ki-67 antibodies (green) (Ki-67(-) cells are marked with the arrows). Nuclei are counterstained with DAPI (blue). (**d**) Representative image of a SA-β-gal-positive cell (2000 mGy, 48 h) marked with an arrow; cytoplasm is colored in dark green-blue. Nuclei are counterstained with Hoechst 33342 (light blue). (**f**) Representative microphotograph of cells immunocytochemically labeled with LC3II (2000 mGy, 48 h). LC3II-positive cell with punctate LC3II (red) is marked with the arrow. Nuclei are counterstained with DAPI (blue).

The proportions of Ki-67-negative cells in control and irradiated fibroblast populations are shown in Figure 3a. The Ki-67 protein is a marker of cell proliferation and is involved in ribosome biogenesis, heterochromatin organization, and mitotic chromosome segregation. It is expressed during interphase (peaking in the S and G2 phases) and mitosis (M) [35,36] but is nearly absent in quiescent and senescent cells [36,37].

There was a statistically significant increase in Ki-67-negative (Ki-67(-)) cells compared to the control only after irradiation with a dose of 2000 mGy (Figure 3a). The representative microphotographs of immunocytochemically stained for Ki-67 irradiated fibroblasts (2000 mGy, 48 h) are shown in Figure S8. The proportion of Ki-67(-) cells increased with longer incubation times (from 24 to 72 h), with the highest relative increase observed in control and 100 mGy irradiated cell populations, likely due to contact inhibition, which would have decreased cell proliferation during 2D cell growth (Figure 3a).

Changes in the proportion of senescence-associated β-galactosidase-positive (SA-β-gal(+)) cells are shown in Figure 3c. This enzyme is commonly used as a marker of cellular senescence [38,39]. A significant increase in SA-β-gal(+) cells was observed only after irradiation with a dose of 2000 mGy (Figure 3c). There was also an increase in the proportion of SA-β-gal(+) cells in the control (statistically insignificant trend) and 100 mGy irradiated cell populations with increasing incubation time (from 24 to 72 h) (Figure 3c). These results are explained by the fact that β-galactosidase is not only a hallmark of senescence but also contact inhibition, since it is also associated with the activation of lysosomes [40]. 

Autophagic (LC3II-positive (LC3II(+))) cells in control and irradiated populations were analyzed (Figure 3e). During autophagy, the cytosolic form of LC3 (LC3I) conjugates with phosphatidylethanolamine to form LC3II, which integrates into autophagosome membranes [41]. Immunocytochemical staining of LC3II allows for the visualization of autophagosomes as granular compartments in the cytoplasm and thus enables the differentiation of autophagic cells. An increase in LC3II(+) cells was observed only after irradiation with 2000 mGy at 24 and 48 h post irradiation (Figure 3e). 

Overall, X-ray irradiation of cultured fibroblasts at 100 mGy did not lead to a statistically significant increase in the proportion of quiescent, senescent, or autophagic cells 24–72 h post irradiation. 

### 2.2. Late Effects

Understanding the long-term effects of ionizing radiation on normal cell populations is crucial. Various mechanisms, such as transgenerational damage transmission and de novo generation due to radiation-induced genomic instability, contribute to long-term effects. This can lead to the premature loss of proliferative capacity or oncotransformation in the progeny of irradiated cells. This study assessed the molecular and cellular effects during the 5th, 10th, and 15th passages post irradiation with doses of 100 and 2000 mGy.

#### 2.2.1. DNA Repair Foci in Later Passages

**Figure 4:**
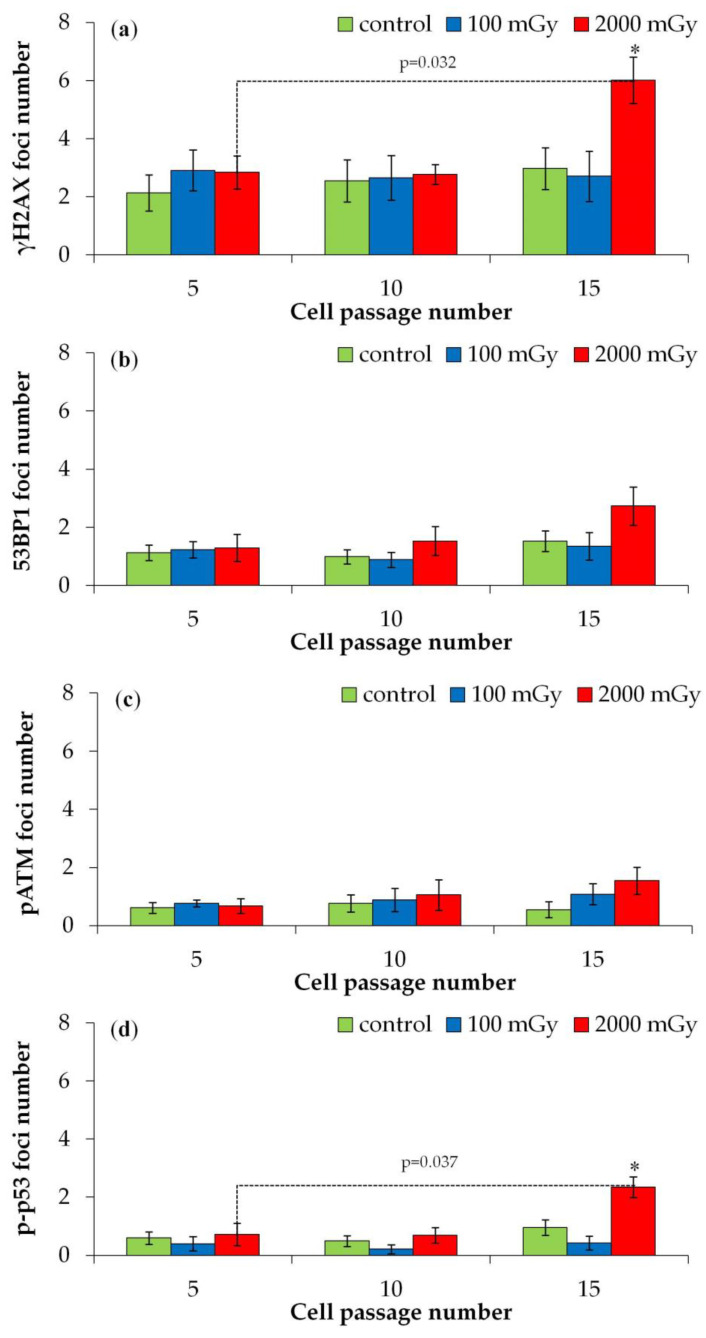
Changes in the number of DNA repair foci depending on the post-irradiation passage number in the control and X-ray-exposed fibroblasts: (**a**) γH2AX; (**b**) 53BP1; (**c**) pATM; and (**d**) p-p53 (Ser-15). * *p* < 0.05 compared with corresponding control.

The number of foci during the 5th, 10th, and 15th passages post irradiation is shown in Figure 3. No significant changes in foci counts were observed in the 5th and 10th passages compared to the control. However, a significant increase in γH2AX and p-p53 foci was noted during the 15th passage only after irradiation with 2000 mGy (Figure 4a,d). 

Overall, these results indicate that irradiation at a dose of 100 mGy does not lead to a statistically significant increase in the number of γH2AX, 53BP1, pATM, and p-p53 (Ser-15) foci, at least during the 15th cell passage after irradiation. A different picture was observed after irradiation at a dose of 2000 mGy. The absence of statistically significant effects during the 5th and 10th passages and the greater number of γH2AX and p-p53 (Ser-15) foci during the 15th passage are apparently due to de novo foci generation as a result of premature cellular aging or radiation-induced genomic instability.

#### 2.2.2. Proliferation, Senescence, and Autophagy in Later Passages 

**Figure 5:**
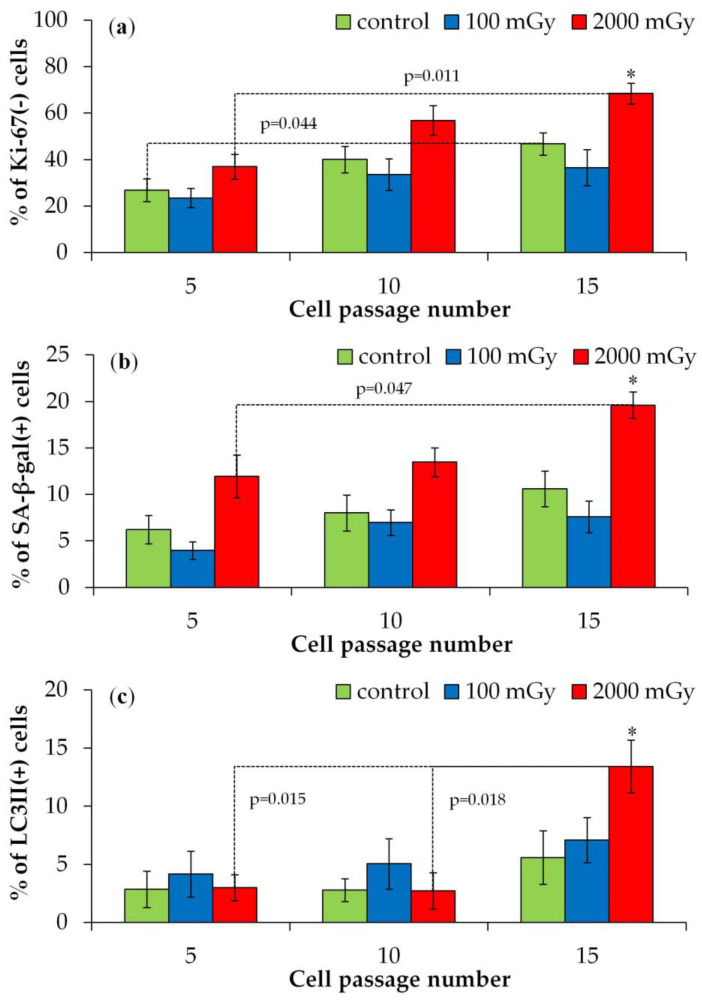
Changes in the proportions of (**a**) Ki-67(-), (**b**) SA-β-gal(+), and (**c**) LC3II(+) cells depending on the post-irradiation passage number in the control and X-ray-exposed fibroblasts. * *p* < 0.05 compared with corresponding control.

The proportions of quiescent (Ki-67-negative, Figure 5a), senescent (SA-β-gal-positive, Figure 5b), and autophagic (LC3II-positive, Figure 5c) cells during the 5th, 10th, and 15th passages post irradiation are shown in Figure 5. Irradiation with 100 mGy did not cause statistically significant changes in these indicators. Additionally, slight tendencies toward a higher proliferative activity and a lower proportion of senescent cells were noted in 100 mGy irradiated populations compared to the control. After irradiation with 2000 mGy, statistically significant increases in quiescent, senescent, and autophagic cells were observed only during the 15th passage compared to the control and the 5th passage (Figure 5). 

## 3. Discussion

It is important to note that two different X-ray regimes were used to irradiate the cells: 100 mGy (100 kVp, 40 mGy/min) and 2000 mGy (200 kVp, 850 mGy/min). This approach has both advantages and disadvantages. On one hand, this made it possible to irradiate the cells for a comparable time period: 150 s for 100 mGy and 141 s for 2000 mGy. It is well known that irradiation time is one of the most important parameters that determines the severity of radiobiological effects [42,43]. On the other hand, the energy of X-ray radiation at a dose of 100 mGy is lower than at a dose of 2000 mGy. The relative biological effectiveness of ionizing radiation depends on the radiation energy. However, in our particular case, these differences were minor and could not elucidate the reported variances in outcomes. The relative biological effectiveness (RBE) of 100 kVp X-ray radiation based on the number of DNA DSBs is only 1.15–1.20 times higher compared to the standard 200 kVp X-ray radiation [44,45]. The study showed that 30 min after irradiation with 100 mGy, the yield of γH2AX, 53BP1, and pATM foci per dose unit and the relative integrated fluorescence of γH2AX, 53BP1, and pATM foci per dose unit were more than twice as high as after irradiation with 2000 mGy. Such results cannot be fully explained by the increased RBE of 100 kVp X-ray radiation used for irradiation at a dose of 100 mGy. The reduction in foci yield at 2000 mGy may reflect biological processes such as the fusion of individual foci into larger “repair centers” or an increase in the speed of DNA repair at the expense of accuracy:

(1) The fusion of individual foci to form larger “repair centers”. The existence of repair centers was demonstrated in the work of T. Neumaier et al. and was used by the authors to explain a similar reduction in the quantitative output of DNA repair foci with increasing radiation dose [46].
(2) It has been shown that with increasing radiation dose, the contribution of the fast mechanism of non-homologous end joining increases [47].

Imperfections in 2D microscopy might also underestimate foci numbers at high foci densities [48], but this is unlikely based on the similar results for the integrated fluorescence of DNA repair foci. 

Interestingly, if the foci yield per dose is not calculated, the results obtained 30 min after irradiation can also be fitted well by a linear model (Figure S9).

The kinetics of post-irradiation changes in DNA repair foci fit a bi-exponential model [49]. It has been shown that in fibroblasts irradiated with X-rays at a dose of 1000 mGy, approximately 50% of the foci are eliminated within the first 4 h after irradiation, followed by a slow elimination over the next 20 h [50]. Only ~5–10% of foci remain uneliminated 24 h after irradiation [39]. No significant differences in the elimination kinetics of γH2AX, 53BP1, and pATM foci were observed between low and high doses in our study. Also, no significant increase in foci was noted at 24 h post irradiation at 100 mGy. 

The small dose of radiation did not increase p-p53 (Ser-15) foci at any studied time point, likely due to the role of p53 in the cell response to radiation. Phosphorylation of p53 at Ser-15 activates G1/S cell cycle arrest at high doses [51,52]. Low doses up to 50–100 mGy do not affect p53 activation in the most sensitive bone marrow cells [53], but low-dose radiation may stimulate fibroblast proliferation [54,55].

This study also analyzed the proportion of quiescent, senescent, and autophagic cells 24–72 h post irradiation. Low-dose radiation did not significantly change these proportions. 

Interesting results were obtained in the study of late effects in the progeny of irradiated cells. A greater number of γH2AX and p-p53 (Ser-15) foci, as well as more SA-β-gal-positive and autophagic cells, was noted in the 15th passage post irradiation at 2000 mGy. X-ray radiation at high doses is known to shorten telomere length in irradiated fibroblasts [56]. With prolonged passaging, this can manifest as a reduction in the Hayflick limit, with a greater number of γH2AX foci in telomeric repeats serving as a marker of senescence [57]. The increase in the number of senescent cells in the 15th passage post irradiation at 2000 mGy was accompanied by an increase in autophagic cells. It was shown that autophagy in prematurely senescent cells is activated via the AMPK pathway [58]. Autophagy and senescence share characteristics and may jointly eliminate cells affected by external stressors such as ionizing radiation and chemical agents and internal forms of stress such as telomere shortening and oncogene activation. However, the role of autophagy in the regulation of cellular senescence is still under debate [59]. Studies of oncogene activation in normal fibroblasts have shown that autophagy and aging are closely linked but not necessarily interdependent [60]. Specifically, autophagy inhibition delays but does not abolish cellular aging [60]. It is also possible that these processes are associated with radiation-induced genomic instability.

An important goal of this study was to assess the long-term effects of low-dose X-ray radiation on populations of fibroblasts. There are known cases of patients refusing diagnostic X-ray examinations due to radiophobia. Therefore, such studies are not only scientifically but also socially significant. It has been demonstrated that exposure to a low dose (100 mGy) of radiation does not induce genomic instability in relation to the studied parameters (DNA repair protein foci) and does not lead to a decrease in proliferative activity and premature aging, at least up to the 15th passage after irradiation.

In conclusion, this study advances the understanding of early and late molecular and cellular effects in human fibroblasts exposed to X-rays during diagnostic procedures and cancer therapy. This knowledge is essential for identifying molecular targets, developing radiation protection strategies, and refining permissible dose limits. 

## 4. Materials and Methods

### 4.1. Cell Culture

Human dermal fibroblasts (Cell Applications, San Diego, CA, USA, catalog number: 106K-05a) were cultured in DMEM culture medium with high glucose content (4.5 g/L) (Thermo Fisher Scientific, Waltham, MA, USA) supplemented with 2 mmol/L L-glutamine (Thermo Fisher Scientific, Waltham, MA, USA), 100 U/mL penicillin, 100 µg/mL streptomycin (Thermo Fisher Scientific, Waltham, MA, USA), and 10% fetal bovine serum (Thermo Fisher Scientific, Waltham, MA, USA). Cell cultivation was performed under the standard conditions of a CO_2_ incubator (37 °C, 5% CO_2_, saturated humidity).

### 4.2. Irradiation

Cells were irradiated in the exponential growth phase (cell population density ~60) using a RUB RUST-M1 X-ray unit (Diagnostika-M LLC, Moscow, Russia) at a dose of 100 mGy (dose rate of 40 mGy/min, voltage 100 kV, anode current of 0.8 mA, 1.5 mm Al filter) or 2000 mGy (dose rate of 850 mGy/min, voltage of 200 kV, anode current of 5.0 mA, 1.5 mm Al filter). A DRK-1-K01 ionization dosimeter (SPC Doza, Zelenograd, Russia) and GAFChromic EBT3 dosimetry film (Ashland Advanced Materials, Bridgewater, NJ, USA) were used to estimate the absorbed dose. Accuracy of dosimetry was around 10%.

To study the early effects of irradiation (up 72 h), cells were seeded at a density of 1 × 10^5^ cells/mL in 2.5 mL of culture medium on coverslips (Thermo Fisher Scientific, Waltham, MA, USA) that were placed in 35 mm Petri dishes (Corning, New York, NY, USA) and incubated under the standard conditions of a CO_2_ incubator for 24 h before irradiation.

To study late effects (5, 10, and 15 cell passages), 2 × 10^6^ cells in T75 culture flasks (Corning, New York, NY, USA) were irradiated. Control and irradiated cells were passaged synchronously every 3 days. The only exclusion was cells exposed to the high dose. Starting from the 12th passage, a significant slowdown in the proliferative activity of cells irradiated at a dose of 2000 mGy was noted. To achieve the same confluence with the control, it took 4 days before the 13th passage, 5 days before the 14th passage, and 7 days before the 15th passage.

Three independent experiments were performed using the same cell culture. Each experiment had three parallel replicates. 

### 4.3. Immunocytochemistry 

Immunocytochemical staining of cells was performed according to a previously described protocol [39]. The following primary antibodies were used: rabbit monoclonal antibodies against γH2AX (phospho S139) (dilution 1:800, clone EP854(2)Y, Abcam, Waltham, MA, USA); mouse monoclonal antibodies against 53BP1 (dilution 1:400, clone BP13, Merck-Millipore, Burlington, VT, USA); mouse monoclonal antibodies against phosphorylated ATM (phospho S1981) protein (dilution 1:200, clone 10H11.E12, Abcam, Waltham, MA, USA); mouse monoclonal antibodies against phospho-p53 (Ser15) (16G8) (dilution 1:400, Cell Signaling Technology, Danvers, MA, USA); mouse monoclonal antibodies against Ki-67 protein (dilution 1:400, clone Ki-S5, Merck-Millipore, Burlington, VT, USA); rabbit polyclonal antibody against LC3BI/II proteins (dilution 1:400, cat. no. ABC929, Merck-Millipore, Burlington, VT, USA). The following secondary antibodies were used: goat anti-mouse IgG H&L (Alexa Fluor 488 conjugated, dilution 1:1600; Abcam, Waltham, MA, USA) and goat anti-rabbit IgG H&L (Alexa Fluor^®^ 555, dilution 1:1600; Abcam, Waltham, MA, USA). Cells were imaged using a Nikon Eclipse Ni-U microscope (Nikon, Tokyo, Japan) equipped with a ProgRes MFcool high-resolution camera (Jenoptik AG, Jena, Germany) using filter sets UV-2E/C, B-2E/C, and Y-2E/C and 60× (foci analysis) or 20× (Ki-67 and LC3II analysis) objectives. For each independent experimental data point, 300–400 cells were analyzed (a total of 900–1200 cells for each summarized data point). Foci were enumerated using DARFI software (http://github.com/varnivey/darfi; accessed on 19 September 2016) and confirmed by manual scoring. The representative screenshots of DARFI program proceeded pictures are shown in Figures S5–S7. Ki-67-negative cells and LC3II-positive cells were counted manually. Cells containing at least ten LC3II punctates were scored as LC3II positive.

### 4.4. Analysis of Senescence-Associated β-Galactosidase-Positive Cells

To quantify the proportion of senescence-associated β-galactosidase-positive (SA-β-gal+) cells, the commercial kit “Cellular Senescence Assay” (EMD Millipore, Burlington, VT, USA, catalog number: KAA002) was used. Cells were stained according to the manufacturer’s protocol, with minor modifications described previously [61]. Stained cells were imaged using an Olympus CKX 41 SF inverted fluorescence microscope (Olympus, Tokyo, Japan) equipped with an Infinity 3-1 camera (Lumenera Copr., Ottawa, ON, Canada) and a 20× objective. The proportions of SA-β-gal+ cells were counted manually. A total of 900–1200 cells for each data point summarized in the three experiments were analyzed. 

### 4.5. Statistical Analysis

Statistical and mathematical data analyses were performed using Statistica 8.0 software (StatSoft, Tulsa, OK, USA). Results are presented as the means of three independent experiments ± standard error (SE). Student’s *t*-test and Mann–Whitney U-test were used to test statistical significance.

## Data Availability

The data used to support the findings of this study are available from the corresponding authors upon request.

## Supplementary Materials

The supporting information can be downloaded at https://www.mdpi.com/article/10.3390/ijms25158253/s1.
