# Supplementary material for: Early and Late Effects of Low-Dose X-ray Exposure in Human Fibroblasts: DNA Repair Foci, Proliferation, Autophagy, and Senescence
Source: Int J Mol Sci. 2024 Jul 28;25(15):8253. doi: 10.3390/ijms25158253 (PMC11311499; doi:10.3390/ijms25158253)
Supplement: Supplementary file 1 [file ijms-25-08253-s001.zip › ijms-3069672-supplementary.pdf]

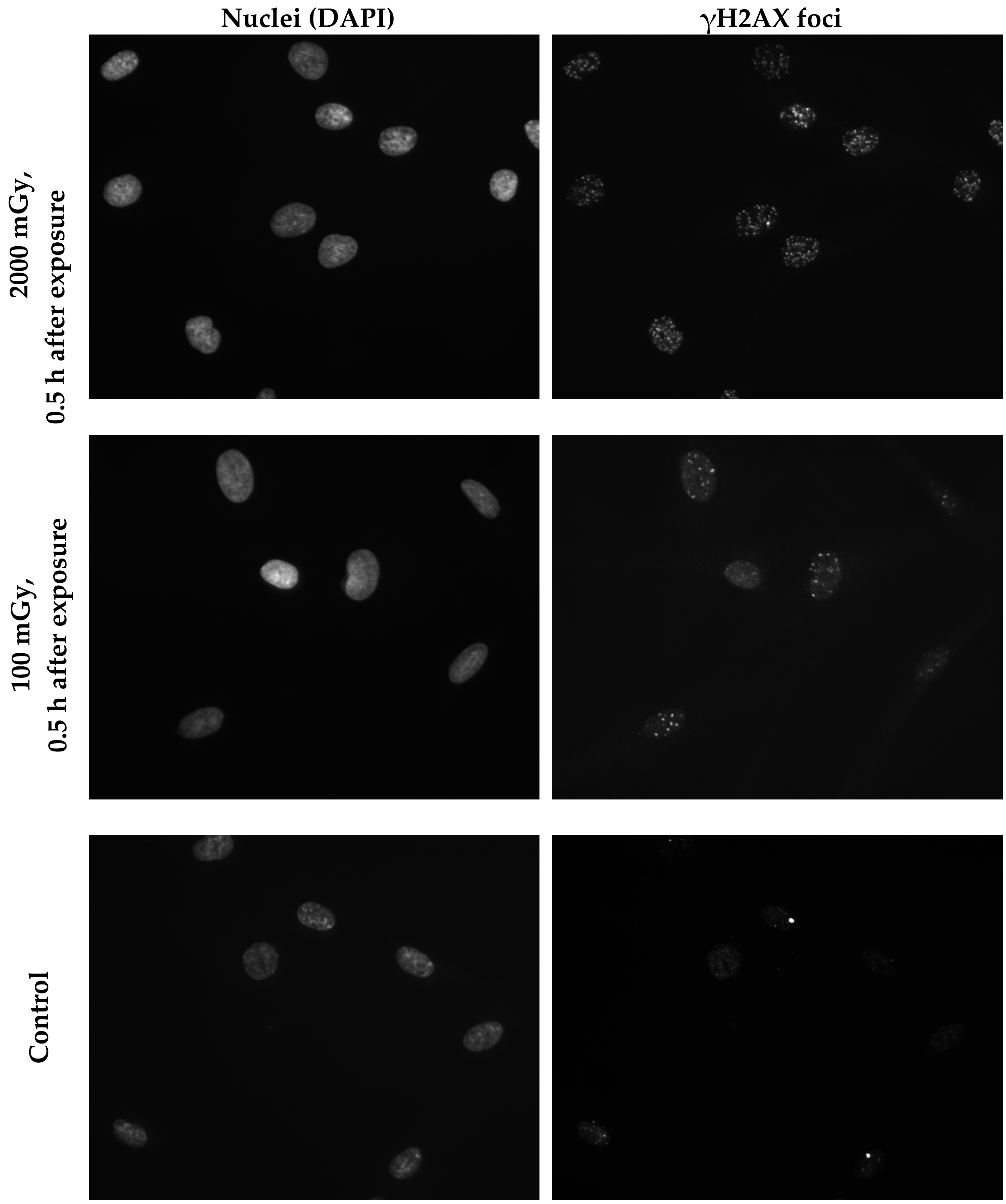

Figure S1. Representative raw microphotographs of immunocytochemically stained for γH2AX control and irradiated fibroblasts.

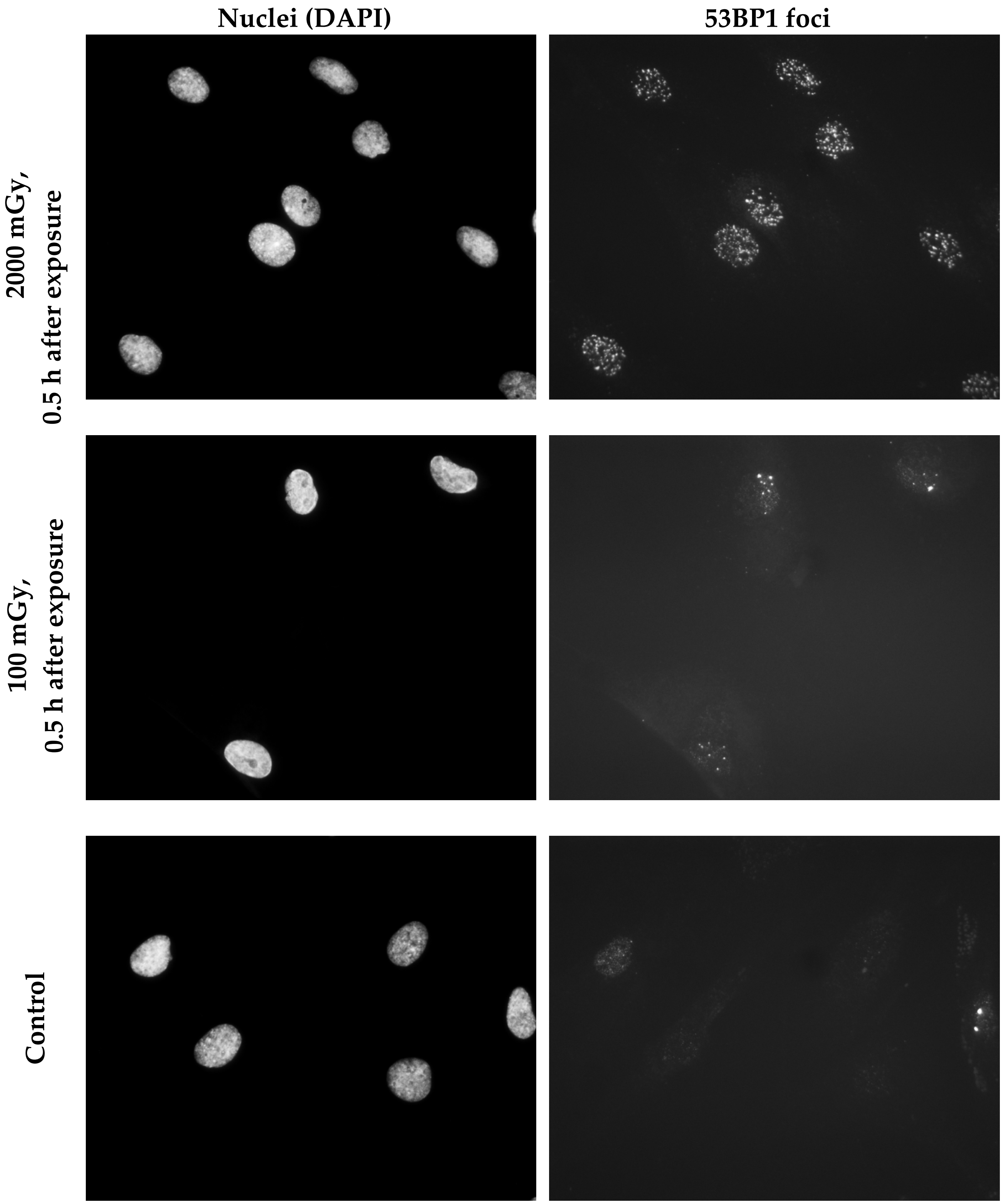

Figure S2. Representative raw microphotographs of immunocytochemically stained for 53BP1 control and irradiated fibroblasts.

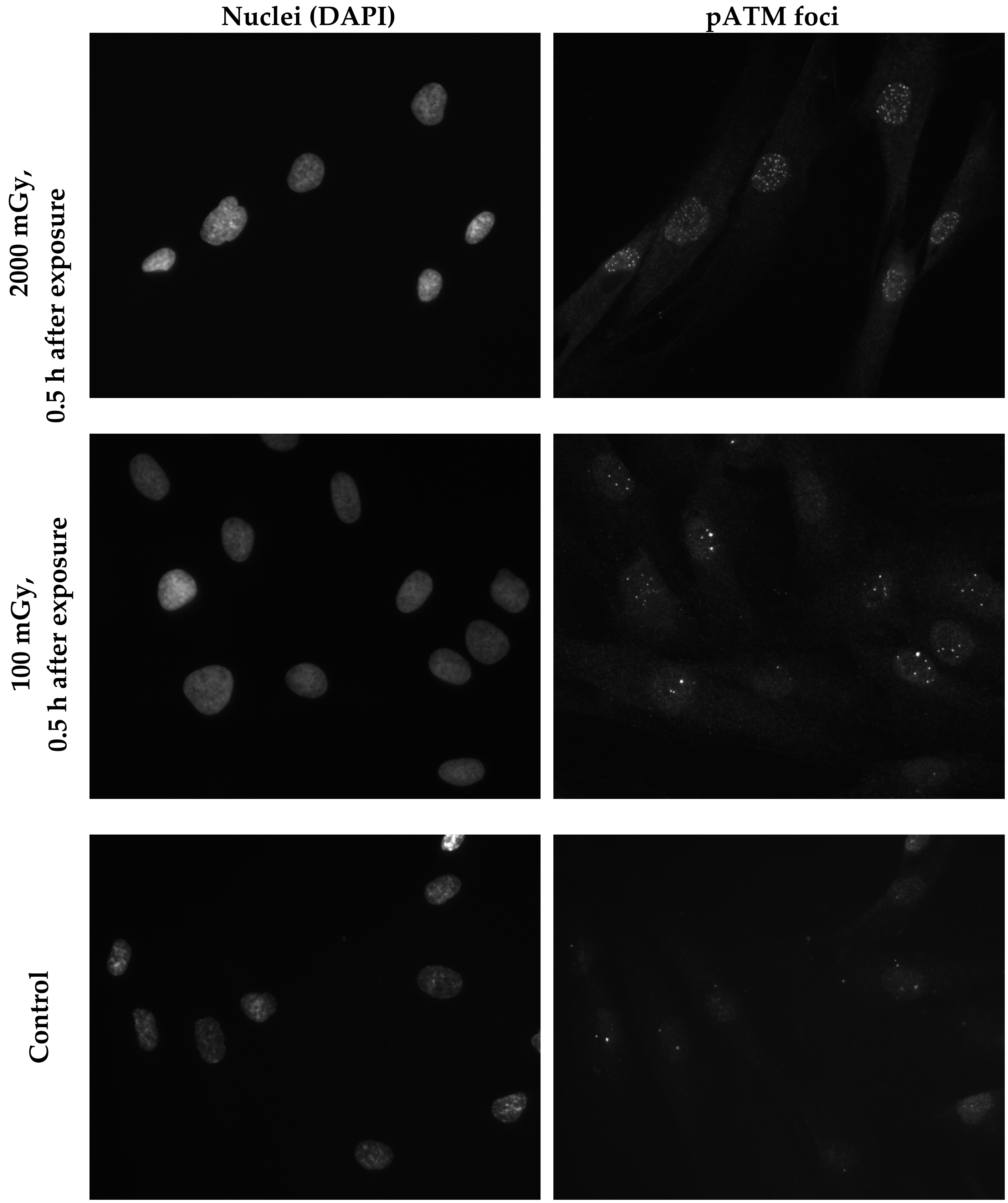

Figure S3. Representative raw microphotographs of immunocytochemically stained for pATM control and irradiated fibroblasts.

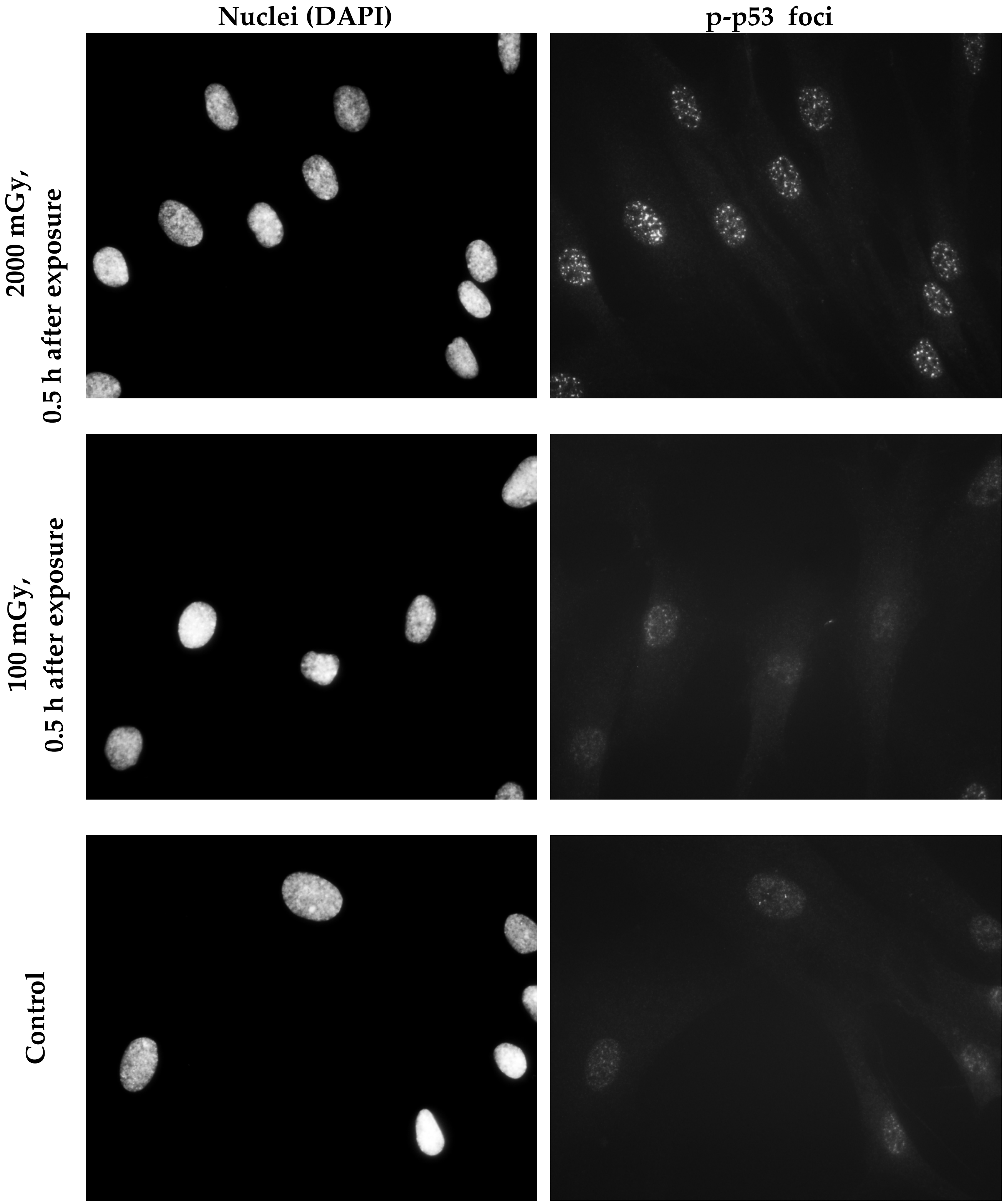

Figure S4. Representative raw microphotographs of immunocytochemically stained for p-p53 control and irradiated fibroblasts.

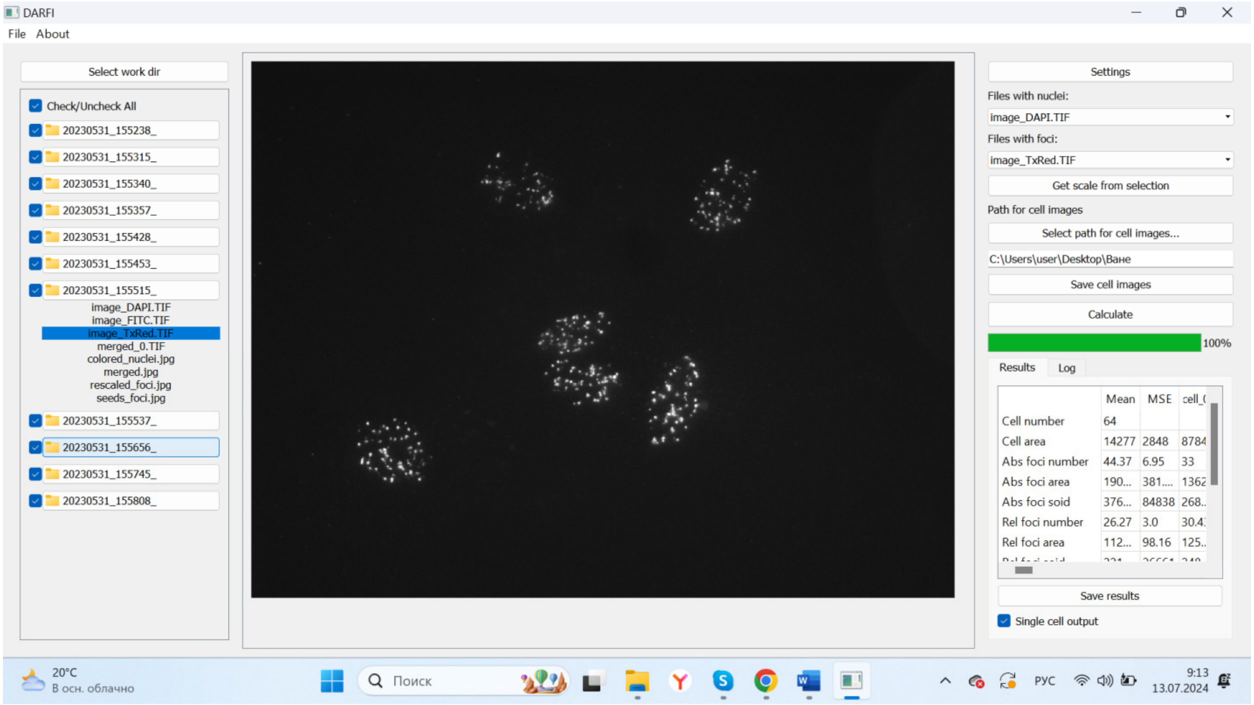

(a)

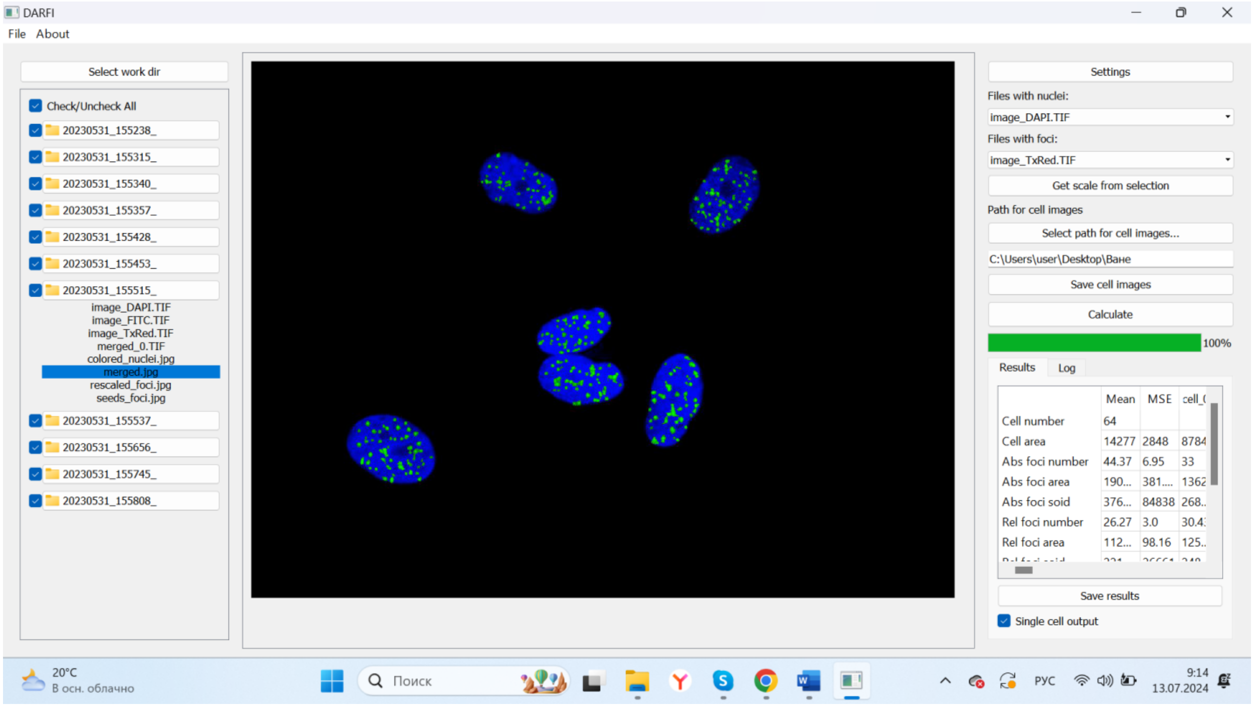

(b)

Figure S5. Representative screenshots of DARFI program proceeding picture of 2000 mGy irradiated fibroblasts (0.5 hours after irradiation) stained for γH2AX: (**a**) untreated original picture, (**b**) proceeded picture with recognized and scored foci. Nuclei colored in blue. Recognized foci colored in green.

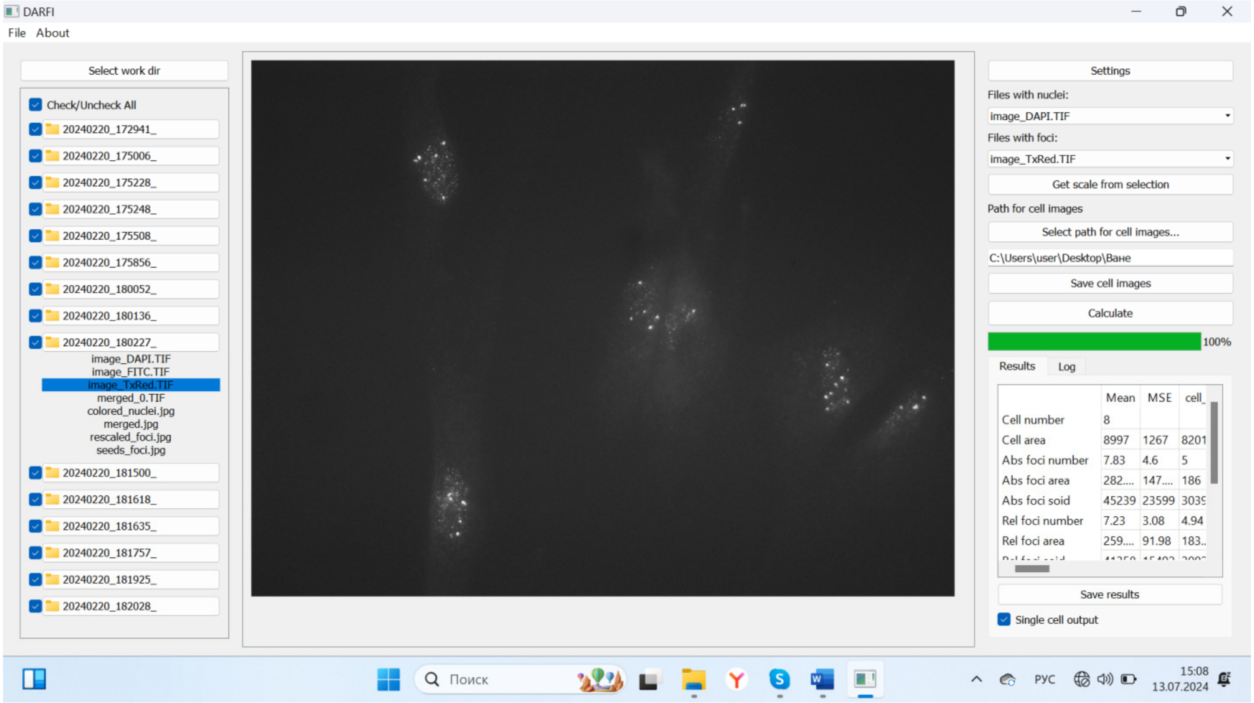

(a)

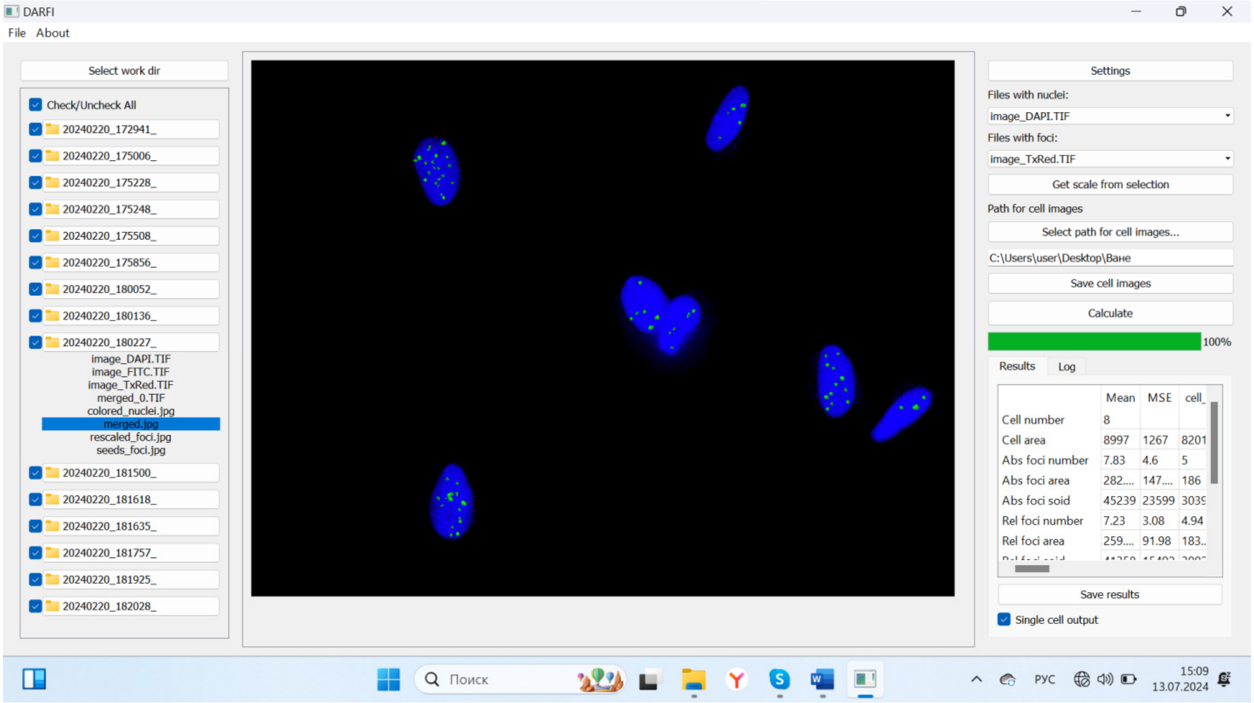

(b)

Figure S6. Representative screenshots of DARFI program proceeding picture of 100 mGy irradiated fibroblasts (0.5 hours after irradiation) stained for γH2AX: (**a**) untreated original picture, (**b**) proceeded picture with recognized and scored foci. Nuclei colored in blue. Recognized foci colored in green.

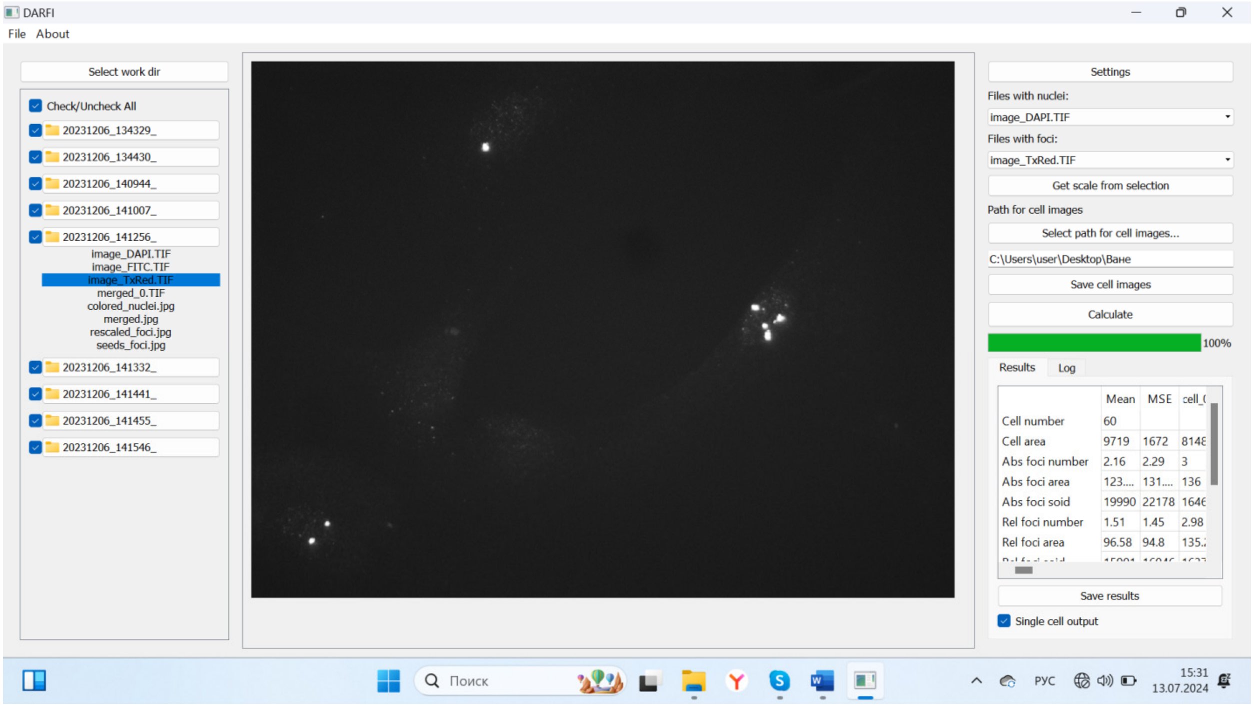

(a)

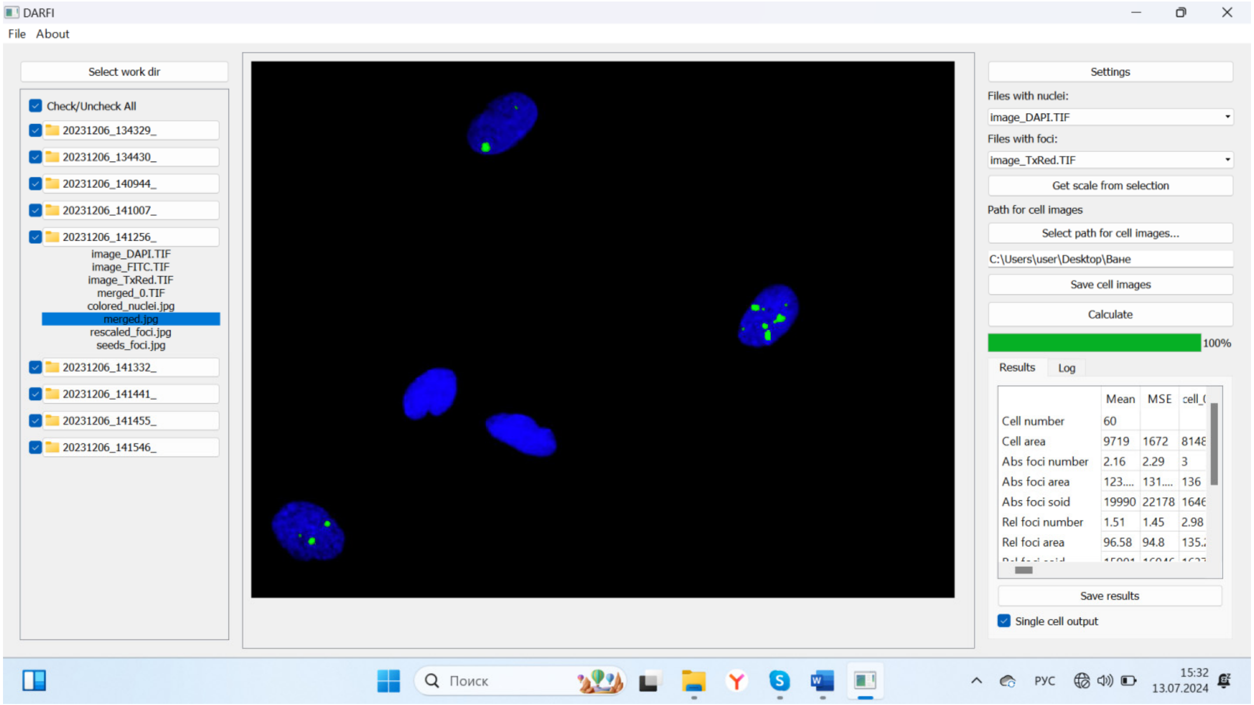

(b)

Figure S7. Representative screenshots of DARFI program proceeding picture of control fibroblasts stained for γH2AX: (**a**) untreated original picture, (**b**) proceeded picture with recognized and scored foci. Nuclei colored in blue. Recognized foci colored in green.

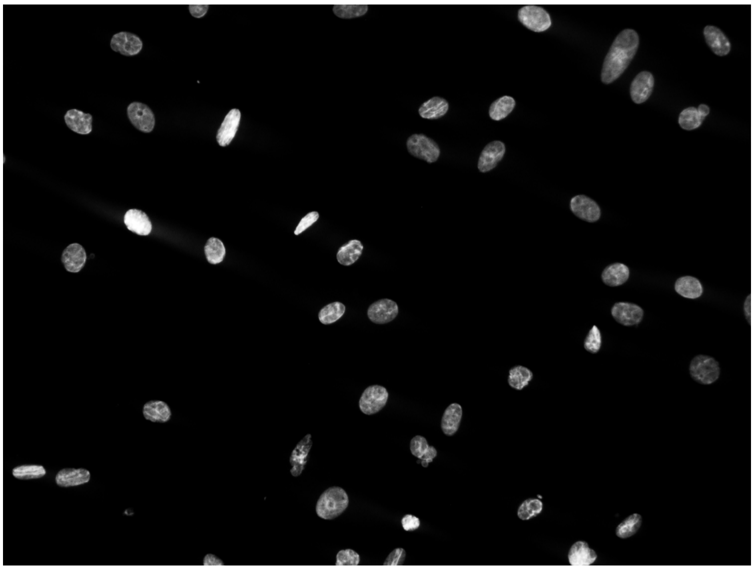

(**a**)

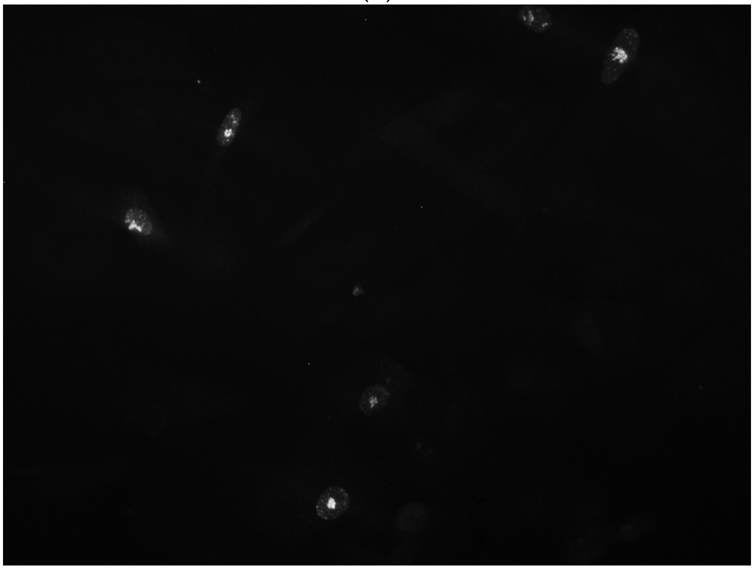

(**b**)

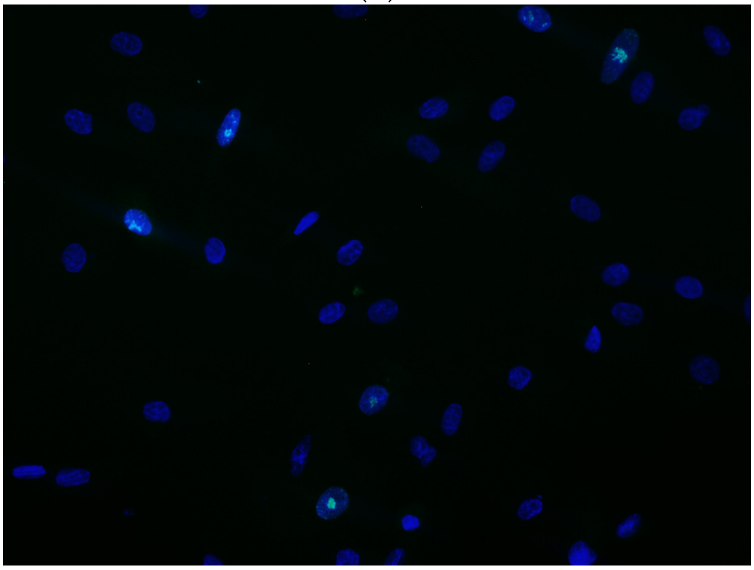

(**a**)

Figure S8. Representative microphotographs of immunocytochemically stained for Ki-67 irradiated fibroblasts (2000 mGy, 48 hours). (**a**) Raw image of DAPI stained nuclei. (**b**) Raw image of Ki-67 stained cells. (**c**) Merged and artificially colored picture. Ki-67 - green. DAPI - blue.

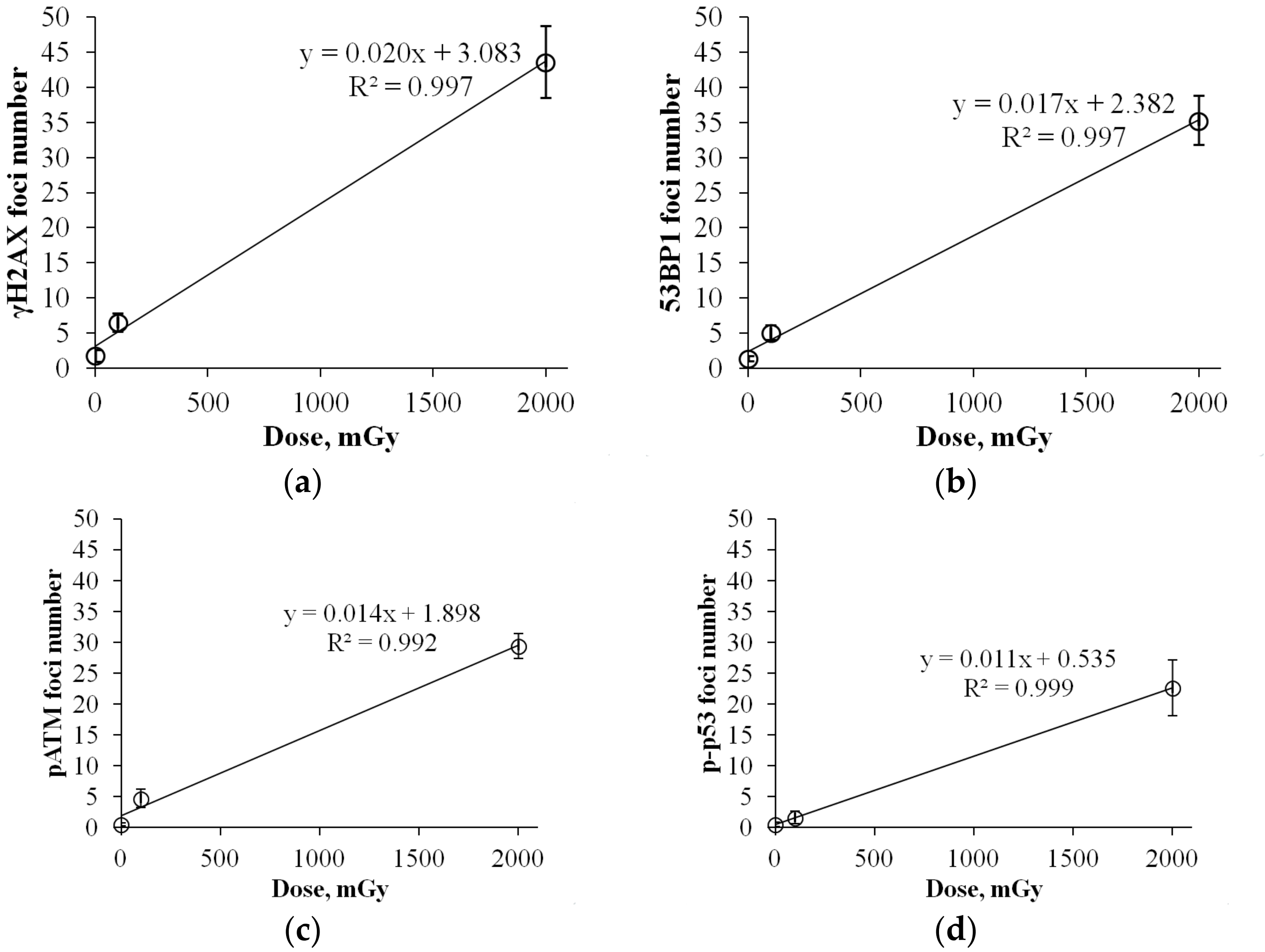

Figure S9. Dose-dependent changes in the foci number in X-ray irradiated human fibroblasts (0.5 hours after irradiation) fitted by linear model: (**a**) γH2AX; (**b**) 53BP1; (**c**) pATM; (**d**) p-p53.
